# Testosterone and anti-Müllerian-hormone (AMH) in lean and overweight male Labrador Retrievers

**DOI:** 10.1186/1751-0147-57-S1-P1

**Published:** 2015-09-25

**Authors:** Elina Andersson, Josefin Söder, Katja Höglund, Ragnvi Hagman, Sara Wernersson, Bodil Ström Holst

**Affiliations:** 1 Department of Clinical Sciences, Swedish University of Agricultural Sciences, Uppsala, Sweden; 2 Department of Anatomy, Physiology and Biochemistry, Swedish University of Agricultural Sciences, Uppsala, Sweden; 3 Centre for Reproductive Biology in Uppsala (CRU), Swedish University of Agricultural Sciences, Uppsala, Sweden

## Introduction

Anti-Müllerian hormone (AMH) and testosterone are produced by the testicles. Both hormones affect sperm production, and hence fertility. In men with a high body condition score (BCS), the testosterone concentration is low.

## Objective

To investigate the influence of body condition score on serum concentration of testosterone and AMH in clinically healthy male dogs. The hypothesis was that overweight dogs have lower testosterone concentrations, and lower or higher concentrations of AMH, than lean dogs.

## Methods

Blood samples from 12 lean (BCS 4-5) and 16 overweight (BCS 6-8) male Labrador Retrievers were analysed. Samples were collected at the same time of the day in all dogs. AMH and testosterone concentrations were analysed with ELISAs (AMH Gen II ELISA, Beckman coulter, and testosterone ELISA, IBL International, GmbH). The Mann-Whitney U test was used for group comparisons.

## Results

Median concentration and inter-quartile range was 9.6 pg/L (7.4-14.1) for AMH and 9.7 nmol/L (6.3-15.4) for testosterone. Three testosterone results were excluded due to technical problems. There were no significant differences between lean and overweight dogs.

## Conclusions

Serum concentrations of testosterone and AMH did not differ between lean and overweight dogs. This could be due to a lack of real difference, that the sample population was too small and the power of the study thus limited, or that the BCS was too low in the overweight dogs (only mild overweight). The concentration of testosterone, and potentially also AMH, varies throughout the day, but due to the standardised sampling, this should not affect the results.

